# Characteristics and phylogenetic analysis of the complete chloroplast genome of *Rosa glomerata* (Rosaceae)

**DOI:** 10.1080/23802359.2022.2107457

**Published:** 2022-09-02

**Authors:** Jiao-Feng Chen, Si-Qi Wang, Hou-Wen Cai, Zhang-Ming Zhu

**Affiliations:** School of Ecology and Environmental Science and Yunnan Key Laboratory for Plateau Mountain Ecology and Restoration of Degraded Environments, Yunnan University, Kunming, China

**Keywords:** *Rosa glomerata*, chloroplast, phylogeny, Rosaceae, high-throughput sequencing

## Abstract

*Rosa glomerata* is a diffuse shrub belonging to *Rosa* sect. *Synstylae*. It is endemic to Southwest China and of high ornamental and economic value. However, its systematic position remains unclear. Here, the complete chloroplast genome of *R*. *glomerata* was assembled using high-throughput sequencing data. The cp genome is 157,064 bp in length with a large single-copy region (LSC) of 86,216 bp, a small single-copy region (SSC) of 18,752 bp, and a pair of inverted repeats (IRs) of 26,048 bp. The overall GC content is 37.2%. A total of 137 genes were annotated, including 90 protein-coding genes, 37 tRNA genes, and eight rRNA genes. Phylogenetic analysis revealed that *R*. *glomerata* is closely related to *R*. *praelucens* of *R*. sect. *Microphyllae* rather than species of *R*. sect. *Synstylae*.

*Rosa glomerata* Rehder and E. H. Wilson (1915), which belongs to *Rosa* L. sect. *Synstylae* DC., is a diffuse shrub endemic to Southwest China at an altitude of 1300–3000 m (Yü et al. 1985). With abundant white flowers and large compound corymbs, *R*. *glomerata* is an important wild rose germplasm resource. However, its phylogenetic position, whether close to *R*. sect. *Synstylae* or other sections (*R.* sect. *Cinnamomeae* DC. ex Ser. and *R*. sect. *Microphyllae* Crep.), is still uncertain because of complex evolutionary history and limited genetic information of DNA markers (Fougère-Danezan et al. 2015; Zhu et al. 2015; Debray et al. 2022). Here, we characterized the complete chloroplast (cp) genome sequence of *R*. *glomerata* and reassessed its phylogenetic position within the genus *Rosa*.

The fresh leaves and specimen of *R*. *glomerata* were collected from Ganluo County, Sichuan, China (102°31′23.22″E, 29°02′47.03″N). *Rosa glomerata* is not an endangered or threatened species and no permission is required to collect specimens. The specimens were identified by Zhang-Ming Zhu and deposited at Herbarium of Yunnan University (http://www.ynu.edu.cn/, Zhang-Ming Zhu, zhangmingzhu@ynu.edu.cn) under the voucher number ZZM1172-6. Total genomic DNA was extracted using a modified CTAB method (Doyle and Doyle 1987). A paired-end library with an insert size of 150 bp was constructed and the library was sequenced using Illumina NovaSeq 6000 platform at Annoroad Gene Technology (Beijing, China). Clean reads were used to assemble the complete cp genome with GetOrganelle v1.7.5 (Jin et al. 2020). Then the cp genome was annotated using PGA (Qu et al. 2019) and reconfirmed by Geneious Prime v2020.0.4 (https://www.geneious.com) based on the reference of *R*. *filipes* Rehd. et Wils. (NC_053856) (Wang et al. 2020).

The cp genome of *R*. *glomerata* (OM519307) is a circular molecule of 157,064 base pairs (bp) presenting a typical quadripartite structure with a pair of inverted repeat regions (IR, 26,048 bp), separated by a large single-copy region (LSC, 86,216 bp) and a small single-copy region (SSC, 18,752 bp). The overall GC content is 37.2%, and the corresponding values in LSC, SSC, and IR regions are35.2%, 31.3%, and 42.8%. The cp genome was annotated with 137 genes, including 90 protein-coding genes, 37 tRNA genes, and eight rRNA genes.

To clarify the systematic position of *R*. *glomerata*, complete cp genome sequences of three outgroup taxa and 18 *Rosa* species representing three subgenera and eight sections (within subgenus *Rosa*) were used to conduct the phylogenetic analysis. Sequences were aligned using MAFFT v7.3 (Katoh and Standley 2013). Then we respectively performed maximum likelihood (ML) analysis with 1,000 bootstrap replicates using RAxML-NG v0.9.0 (Kozlov et al. 2019), and Bayes inference (BI) for 1,000,000 generations with MrBayes v3.2.7 (Ronquist et al. 2012) under the best nucleotide substitution model of GTR + I+G4, which was calculated by ModelTest-NG (Darriba et al. 2020). Phylogenetic relationships resolved in this study are inconsistent with the conventional infrageneric classification (Rehder 1940; Wissemann 2017). *Rosa glomerata* showed a close affinity to *R*. *praelucens* Byhouwer (*R*. sect. *Microphyllae*) and formed a clade sister to species of *R*. sect. *Cinnamomeae*, rather than close to species of *R*. sect. *Synstylae* (Figure 1). The results were consistent with previous phylogenies based on plastid markers (Zhu et al. 2015; Debray et al. 2022). And the cp genome of *R*. *glomerata* will provide valuable information for further studies and revisions of the genus *Rosa*.

**Figure 1.**
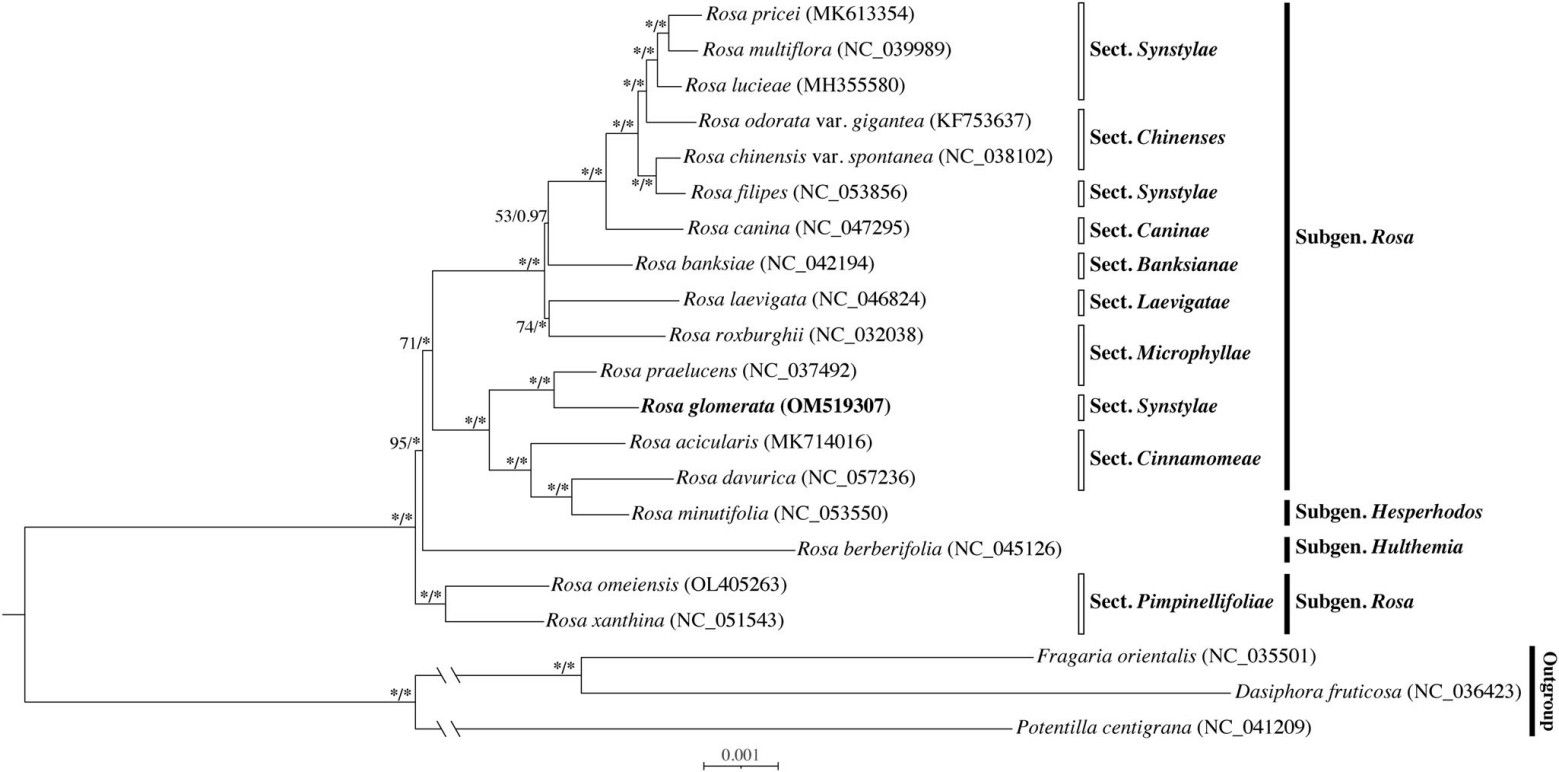
Phylogeny of complete cp genome dataset. ML bootstrap support values (left) and BI posteriori probability (right) are shown before the nodes, ‘*’ indicate 100% support.

## Data Availability

The genome sequence data that support the findings of this study are openly available in GenBank of NCBI at https://www.ncbi.nlm.nih.gov/ under the accession no. OM519307. The associated BioProject, SRA, and Bio-Sample numbers are PRJNA806373, SRR17999625, SAMN25875939, respectively.
